# Six Homeoproteins Directly Activate *Myod* Expression in the Gene Regulatory Networks That Control Early Myogenesis

**DOI:** 10.1371/journal.pgen.1003425

**Published:** 2013-04-25

**Authors:** Frédéric Relaix, Josiane Demignon, Christine Laclef, Julien Pujol, Marc Santolini, Claire Niro, Mounia Lagha, Didier Rocancourt, Margaret Buckingham, Pascal Maire

**Affiliations:** 1 UPMC Paris 06, UMR-S 787, Paris, France; 2 INSERM, Avenir Team, Pitié-Salpétrière, Paris, France; 3 Institut de Myologie, Paris, France; 4 Institut Cochin, INSERM U1016, CNRS (UMR 8104), Université Paris Descartes, Paris, France; 5 Laboratoire de Physique Statistique, ENS, Paris, France; 6 CNRS URA 2375, Department of Developmental Biology, Pasteur Institute, Paris, France; Institut de Recherches Cliniques de Montréal, Canada

## Abstract

In mammals, several genetic pathways have been characterized that govern engagement of multipotent embryonic progenitors into the myogenic program through the control of the key myogenic regulatory gene *Myod*. Here we demonstrate the involvement of Six homeoproteins. We first targeted into a *Pax3* allele a sequence encoding a negative form of Six4 that binds DNA but cannot interact with essential Eya co-factors. The resulting embryos present hypoplasic skeletal muscles and impaired *Myod* activation in the trunk in the absence of *Myf5/Mrf4*. At the axial level, we further show that *Myod* is still expressed in compound *Six1/Six4:Pax3* but not in *Six1/Six4:Myf5* triple mutant embryos, demonstrating that *Six1/4* participates in the *Pax3-Myod* genetic pathway. *Myod* expression and head myogenesis is preserved in *Six1/Six4:Myf5* triple mutant embryos, illustrating that upstream regulators of *Myod* in different embryonic territories are distinct. We show that *Myod* regulatory regions are directly controlled by Six proteins and that, in the absence of Six1 and Six4, Six2 can compensate.

## Author Summary

The onset of skeletal muscle formation is controlled by complex gene regulatory networks. By manipulation of these genetic pathways in the mouse embryo, we have examined the interplay between genes encoding the transcriptional regulator Pax3; the major myogenic determination proteins Myf5, Mrf4, and Myod; as well as genes encoding homeodomain proteins Six1 and Six4. In the absence of Myf5 and Six1/4, *Myod* expression is compromised. We demonstrate that key regulatory elements of the *Myod* gene are directly targeted by Six factors, including Six2, which is unexpectedly upregulated in the absence of Six1 and Six4. This work therefore reveals new aspects of the gene regulatory networks that control myogenesis.

## Introduction

The *Pax-Six-Eya-Dach* genetic network was first identified in *Drosophila* as a key transcriptional regulator of compound eye development. Within this network, the *Pax* gene, *Eyeless*, is an upstream regulator of genes for the Six transcription factor sine oculis and of its co-factor Eyes absent (Eya), with feedback regulation between these genes [1], [2]. Vertebrate homologues are involved in eye development [3] but also in other developmental processes, suggesting that the mechanisms orchestrated by this genetic network are conserved and used for multiple types of organogenesis and tissue specification during embryonic development [4], [5], [6]. Indeed in *Drosophila*, *Pox meso*, *dSix4* and *Eya* are involved in somatic myogenesis [7], [8], [9].

During vertebrate myogenesis, Pax3 and Pax7 are important upstream regulators of myogenic progenitor cell behaviour, survival and fate, as shown by genetic manipulations in the mouse embryo [5], [10]. Skeletal muscles of the trunk and limbs are derived from progenitors present in the dorsal dermomyotome domain of the somites which segment from paraxial mesoderm and mature following an anterior/posterior gradient along the axis of the vertebrate embryo. Pax3 is expressed throughout the epithelial dermomyotome and Pax7 in its central domain that will give rise to the progenitor cells of the myotome [5]. Six1/4, together with the Six co-activators, are also present in the dermomyotome together with Eya1/2, expressed at a high level in the epaxial and hypaxial domains. These *Six* and *Eya* genes have been shown to control the myogenic progenitor cell population, particularly in the hypaxial domain, where Pax3 also plays a key role in the survival and delamination/migration of myogenic progenitors. Interactions between these genes in the myogenic context, were suggested by overexpression experiments in the chick embryo, in somite explants [11] and in cell culture [12]. Analysis of compound *Six1/4* and *Eya1/2* mutants show that these factors regulate *Pax3* in the hypaxial dermomyotome, whereas *Pax3* expression is increased in the posterior dermomyotome in the absence of Six transactivation [13], [14], [15]. In the head muscles, which form from anterior unsegmented paraxial mesoderm, Pax3 is not expressed in myogenic progenitors, and Pax7 only later, whereas Six1 and Eya1 co-factors are present and active [15], [16], [17], as well as Pitx2 which acts as an upstream regulator of craniofacial myogenesis [18].

Entry into the myogenic programme, both in the head and trunk, depends on the myogenic determination factors Myf5/Mrf4 and Myod. Another member of this family of basic-helix-loop helix transcription factors, Myogenin, intervenes at the level of myogenic differentiation [19]. During the onset of myogenesis in the mouse embryo, *Myf5* is expressed before *Myod* and in the absence of Myf5 and Mrf4 the activation of *Myod* is delayed [20]. In *Pax3*;*Myf5/Mrf4* double mutants, *Myod* is not activated and skeletal muscle does not form in the trunk and limbs. In the absence of Pax3, the onset of myogenesis in the epaxial somite, although perturbed [21] takes place, with *Myf5/Mrf4* activation through Wnt, and Shh signalling pathways [22], acting on an early epaxial enhancer of *Myf5*. Later activation of *Myf5* in the hypaxial somite and in myogenic progenitor cells that have migrated to the limb, depends on another enhancer element which is directly regulated by Pax3 [23] and by Six1/4 [24], illustrating the synergistic action of these upstream regulators in driving the expression of myogenic determination genes. Double mutant analyses of *Six1/4* and *Eya1/2* show a reduction in *Myod* expression [15] in the somites during embryonic development and Six binding to *Myod* regulatory elements has been shown in cultured muscle cells [25]. This, together with the absence of *Pax3* expression in the hypaxial somite observed in *Six1/4* and *Eya1/2* mutants, suggests that *Six/Eya* may intervene in the *Pax3/Myod* genetic cascade. *Six/Eya* also, unlike *Pax3*, are expressed in craniofacial myogenic progenitors [17], and play a role in the onset of differentiation, when they directly regulate the activation of *Myogenin* [26] [15], and later when they directly regulate the expression of genes coding for sarcomeric proteins [27], [28].

In this paper, we use genetic tools to further investigate how *Six1/4*, with the participation of *Eya1/Eya2*, intervene in the myogenic hierarchy. By analysis of *Six/Pax* and *Six/Myf5* mutants, together with mutants in which a dominant negative form of the Six coding sequence has been targeted to an allele of *Pax3*, we show that *Six1/4* are essential regulators in the *Pax3/Myod* genetic cascade, revealed in the absence of *Myf5* at the body level. This is confirmed by the demonstration that *Myod* activation depends directly on Six binding to key enhancer sequences upstream of the *Myod* gene, which controls *Myod* expression in all myogenic lineages.

## Results

### *Pax3* and *Six1* act through a common genetic pathway

**Figure 1:**
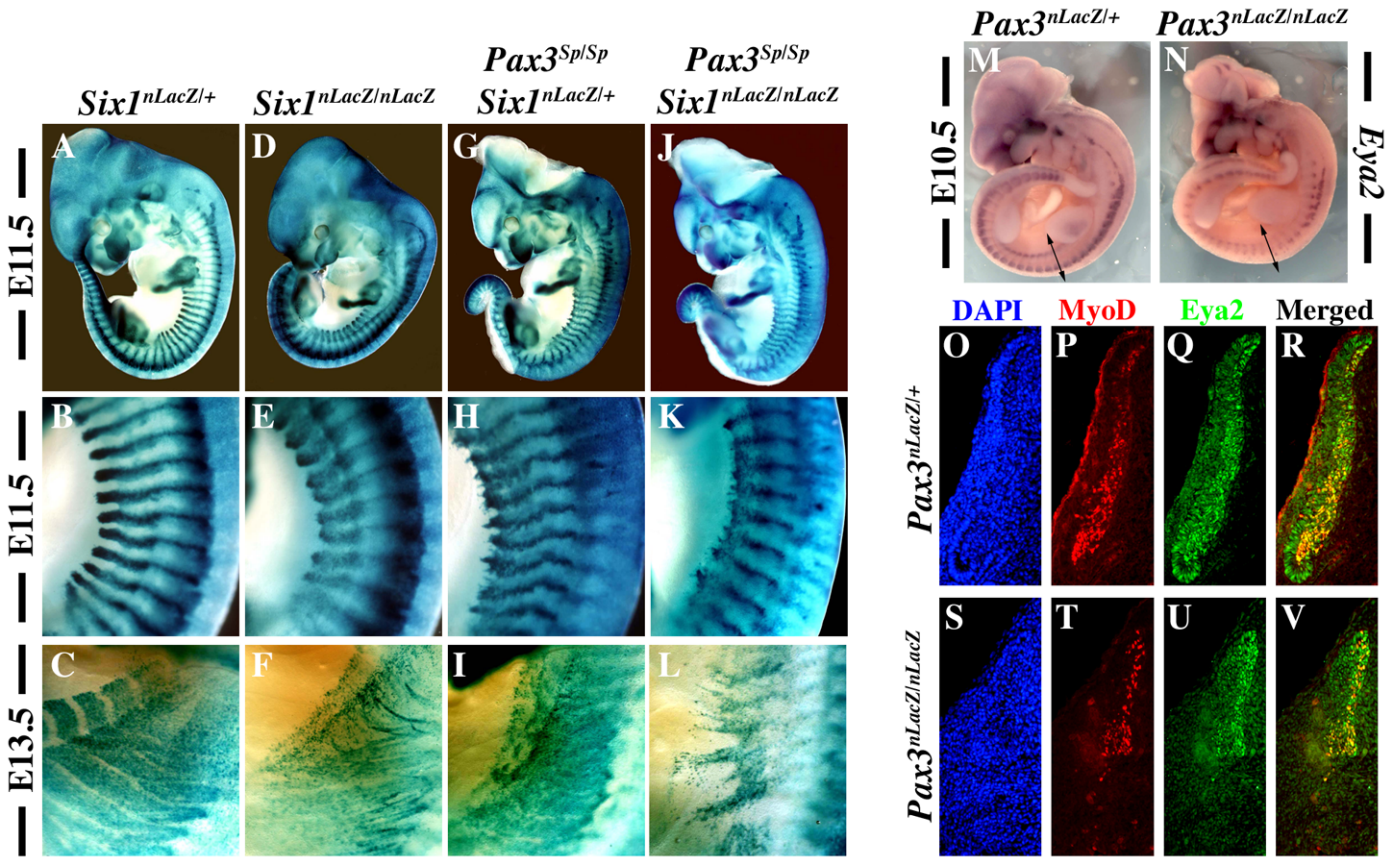
Genetic relationships between *Six1* and *Pax3*. X-Gal staining of *Six1^nLacZ/+^* heterozygous embryos on a wild type background (A–C) and on a *Pax3* mutant background (*Pax3^Sp/Sp^)* (G–I) at E11.5 (A–B, G–H) and E13.5 (C, I) shows that *Six1* expression, followed by the *nLacZ* reporter, is maintained in the absence of Pax3. Comparison of *Pax3^Sp/Sp^* : *Six1^nLacZ/+^* embryos (G–I) with *Six1^nLacZ/nLacZ^* mutants (D–F) shows a reduction of the extent of the somite where *Six^nLacZ^* is expressed, particularly hypaxially at E11.5 (E, H). Disorganisation and loss of hypaxial muscle fibers is observed at E13.5 (F, I) in the interlimb level. These phenotypes are more severe in *Pax3^Sp/Sp^ : Six1^−/−^* double mutants (J,K), notably at E13.5 (L). B,E,H,K and C,F,I,L show enlargements in the interlimb region. M-N, Whole mount *in situ* hybridization using an *Eya2* probe on *Pax3^nLacZ/+^* (M) and *Pax3^nLacZ/nLacZ^* (N) embryos at E10.5 shows that *Eya2* expression is independent of Pax3. O-V, co-immunohistochemistry with Eya2 (Q,R,U,V) and Myod (P,T,R,V) antibodies on interlimb sections of *Pax3^nLacZ/+^* and *Pax3^nLacZ/nLacZ^* embryos confirms continuing expression of Eya2 in the absence of Pax3. Reduction of *Eya2* expression, notably in hypaxial lips of the somites, is due to dermomyotome reduction in the *Pax3* mutant. O,S, DAPI staining.

In order to investigate whether *Pax3* and *Six1* act in the same genetic pathway, we analysed *Pax3/Six1* double mutants. Comparison of *Six1^nLacZ/nLacZ^* and *Pax3^Sp/Sp^* mutant embryos from the same litter shows that somite defects are similar at E11.5 and E13.5 with more cell dispersion of *Six^nlacZ^* cells, notably hypaxially, in the *Pax3* mutant (Figure 1A–1I). The somitic phenotype of *Six1/Pax3* double mutants is similar to that of the *Pax3* mutant, but with more somite truncation at E11.5 (Figure 1J–1K), consistent with partially overlapping function of Pax3 and Six1 at this stage. At E13.5 however, the phenotype of *Pax3*/*Six1* double mutant embryos is clearly more pronounced than either single mutant (Figure 1F, 1I, 1L), indicating that *Pax3* and *Six1* have separate functions at later stages. We had shown already that the expression of *Eya1* and *Eya2* is maintained in *Six1^−/−^* :*Six4^−/−^* mutants, and that the expression of *Eya1* is preserved in the *Pax3* mutant [15]. We now show that this is also the case for *Eya2*, which continues to be transcribed in the myogenic cells still present in somites of the *Pax3* mutant (Figure 1M, 1N, 1Q, 1U). Activation of *Eya1* and *Eya2* is therefore independent of Six1/4 and Pax3. Furthermore, we note that the expression of Myod is only detected in Eya2 expressing cells of the somite, in the absence of Pax3 (Figure 1T, 1U, 1V), consistent with the proposed involvement of Eya co-factors acting with Six1/4, upstream of *Myod* during mouse embryogenesis [15].

### Targeting of a dominant negative form of Six4 (Six4Δ) into the *Pax3* locus

**Figure 2:**
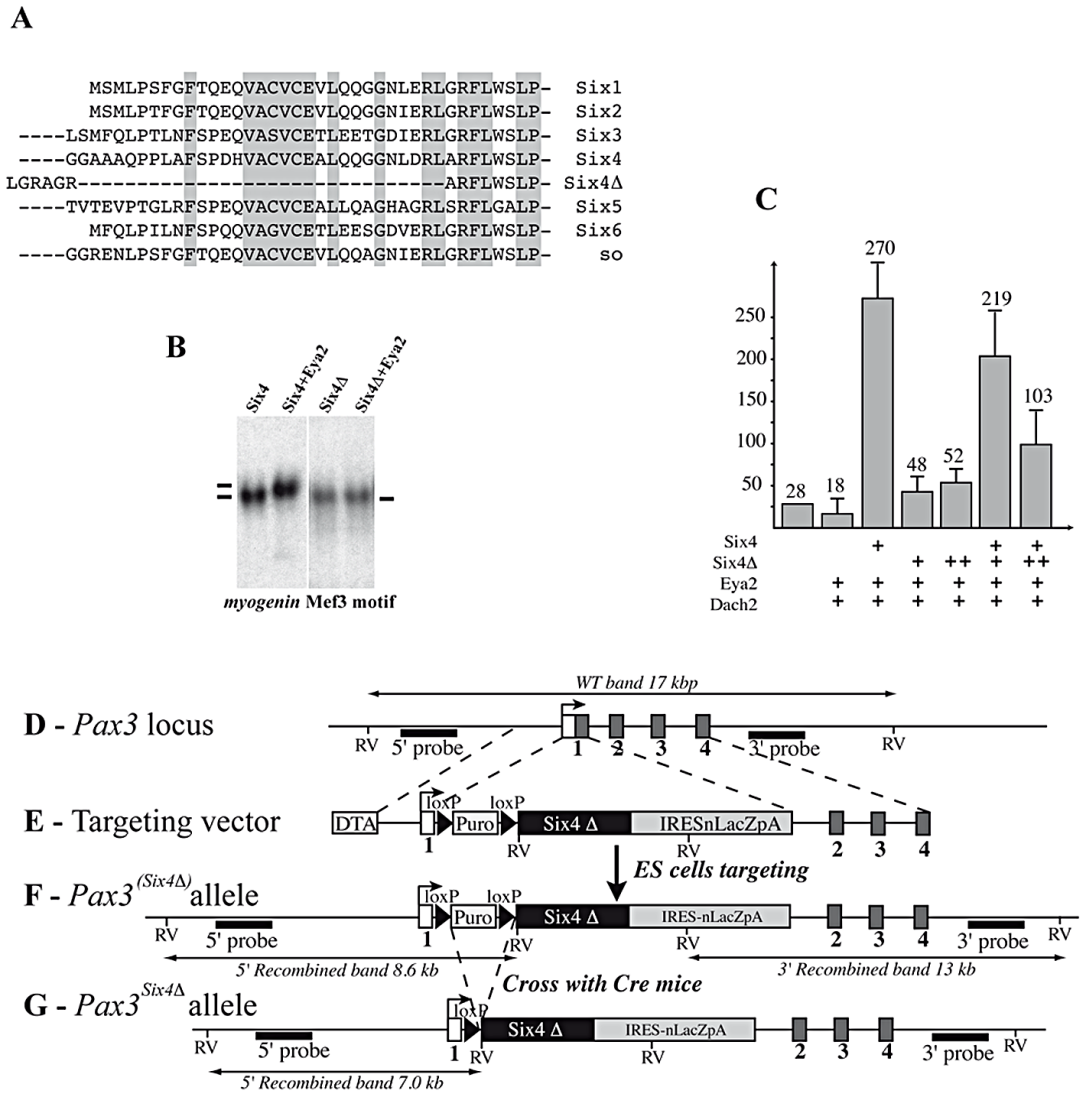
Targeting of a sequence encoding dominant negative Six4 into the *Pax3* locus. A, Alignment of Six protein sequences shows conservation of the N-terminal-most regions of the Six-domain. This region is absent in the Six4Δ mRNA splicing variant. B, Bandshift assays show that Six4 and Six4Δ bind the MEF3 site, but that only Six4 can interact with Eya2 protein to form a larger complex. C, Transfection experiments performed in primary cultures of chick myoblasts show that Six4 and Eya2 synergistically activate transcription of a luciferase reporter driven by the multimerized MEF3 sequence. In contrast, Six4Δ and Eya2 display no functional synergy, and increasing amounts of Six4Δ compete for Six4-Eya2 transcriptional activation. Y axis, ratio between Luciferase and Renilla activities in arbitrary units. D-G, Strategy for targeting the Six4Δ coding sequence into an allele of *Pax3*. The probes and restriction enzymes (EcoRV: RV) are indicated, with the size of the resulting wild-type and recombined restriction fragments. The targeting construct (E) contains 2.4 kb and 4 kb of 5′ and 3′ genomic flanking sequences of the mouse *Pax3* gene. A floxed *puromycin-pA* selection marker (Puro), replaces the coding sequence in exon 1 of *Pax3* (D), followed by a di-cistronic cassette containing the murine *Six4Δ* cDNA comprising the whole coding region, followed by an *IRESnLacZ* cassette and by a final *pA* signal. The *IRESnLacZ* allows easy detection of *Six4Δ* expression [32]. A counter-selection cassette encoding the A subunit of Diptheria Toxin (DTA) was inserted at the 5′end of the vector. After homologous recombination in embryonic stem (ES) cells, *Six4Δ-IRESnLacZ* expression from the *Pax3^(Six4^* ^Δ*-IRESnLacZ)*^ allele is blocked by the floxed *puromycin-pA* cassette (F) and is therefore conditional to removal by crossing with a *PGK*-*Cre* mouse [52]. This generates the *Pax3^Six4Δ-IRESnLacZ^* allele (abbreviated *Pax3^Six4Δ^)* (G).

To investigate the role of the *Pax3*-*Six*-*Eya* network *in vivo* while bypassing both functional compensation between genes in the same family, and potential problems of cell loss due to the function of Pax3 in cell survival, we adopted a dominant negative approach. We selected a Six coding sequence mutated in the Eya interaction domain, but nevertheless able to bind specifically to the Six (MEF3) binding site. Nuclear translocation of the co-activator Eya depends on the Six-Eya interaction which requires the N-terminal Six domain [29], [30], however this domain is also required for DNA binding specificity [31]. We therefore used a sequence encoding an alternative splice variant of Six4, *Six4Δ* (isolated from a mouse muscle cDNA library), which is divergent in the N-terminal-region of the conserved Six binding domain (Figure 2A). The truncated Six protein encoded by *Six4Δ* is still able to bind DNA, but has lost the capacity to associate with Eya2, as shown in gel mobility shift analyses (GMSA) (Figure 2B). While full length Six4 protein synergizes with Eya2 to activate MEF3 reporter activity in transient transfection assays, Six4Δ is unable to synergize with Eya2, and increasing amounts of added Six4Δ competes with the Six4-Eya2 transcription complex, leading to decreased transcriptional activation (Figure 2C).

**Figure 3:**
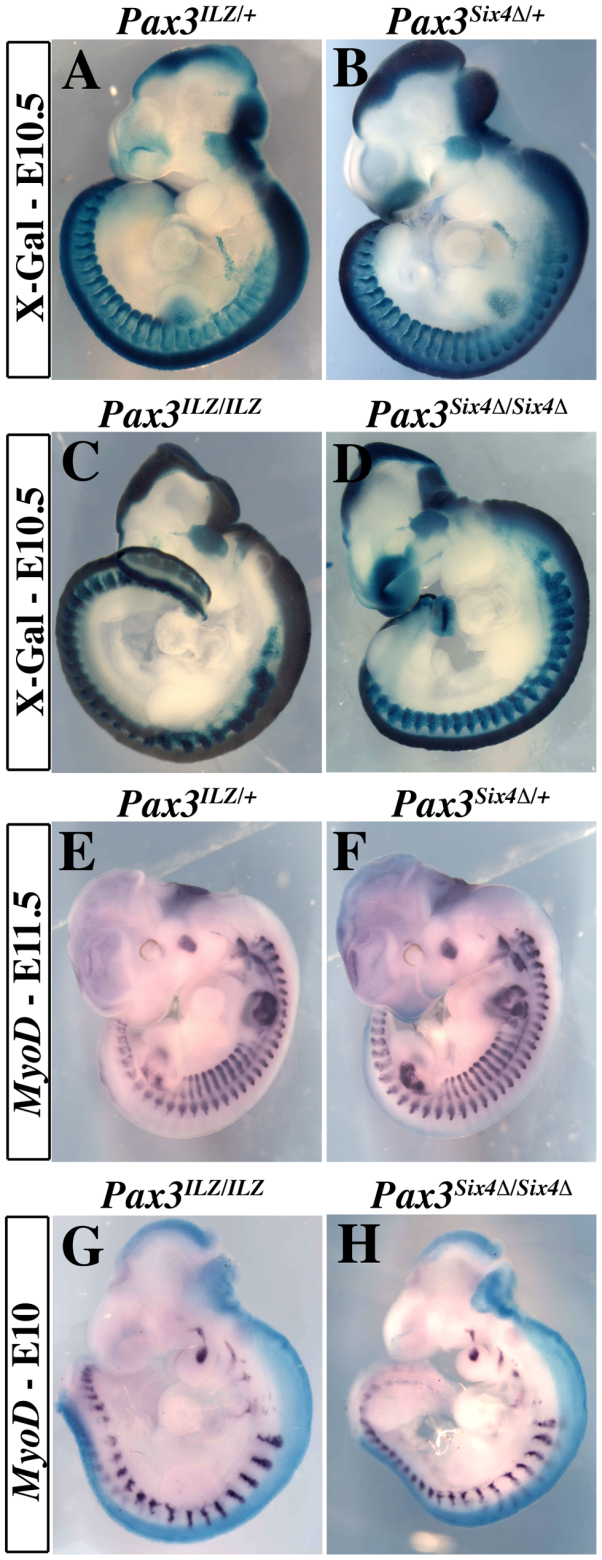
Expression of *Six4Δ* does not perturb normal embryonic development nor rescue *Pax3* mutant deficiencies. A–B, X-Gal staining of *Pax3^IRESnLacZ/+^* (*Pax3^ILZ/+^*, A) and *Pax3^Six4Δ/+^* (B) embryos at E10.5 demonstrates correct expression of the *Six4Δ* transgene. C–D, X-Gal staining of homozygotes *Pax3^ILZ/ILZ^* (C) and *Pax3^Six4Δ/Six4Δ^* (D) embryos at E10.5 demonstrates that the *Six4Δ* sequence does not rescue deficiencies due to the absence of Pax3 (Exencephaly, spina bifida, lack of limb muscles, somitic defects and neural crest cell deficiencies). E–F, Whole mount *in situ* hybridization using a *Myod* probe on *Pax3^ILZ/+^* (E) and *Pax3^Six4Δ/+^* (F) embryos at E11.5 shows that the *Six4Δ* sequence does not overtly perturb *Myod* expression. G–H, Whole mount *in situ* hybridization using a *Myod* probe on homozygote *Pax3^ILZ/ILZ^* (G) and *Pax3^Six4Δ/Six4Δ^* (H) embryos at E10 shows that the onset of *Myod* expression is similar to that of a *Pax3* mutant, in the absence of *Pax3* but in the presence of a dominant negative *Six4Δ* (H).

We targeted the *Six4Δ* sequence into an allele of *Pax3*, to evaluate the function of the Six-Eya interaction during myogenesis *in vivo*. To avoid potential problems of lethality, we used a conditional strategy (see Figure 2D–2G), similar to that previously reported [32], with an *IRESnLacZ* reporter following the *Six4Δ* sequence, to monitor expression. X-Gal staining revealed correct expression of the reporter at E10.5 when compared to embryos where an *nLacZ* reporter is targeted into an allele of *Pax3* (*Pax3^IRESnLacZ/+^*, abbreviated *Pax3^ILZ/+^*) (Figure 3A–3B). This was also the case after *Pax3 in situ* hybridization, compared to wild type (WT) embryos (data not shown, and see [32]). Despite robust *Six4* **Δ** *-IRESnLacZ* expression, these embryos did not present any obvious defects and indeed *Pax3^Six4^* ^**Δ***-IRESnLacZ/+*^ (abbreviated *Pax3^Six4^* ^**Δ***/+*^) mice are viable and fertile.

We went on to test whether expression of *Six4Δ* driven by *Pax3* was able to rescue aspects of the *Pax3* mutant phenotype. X-Gal staining of *Pax3^ILZ/ILZ^* or *Pax3^Six4Δ/Six4Δ^* homozygote embryos at E10.5 (Figure 3C–3D) showed the same defects previously reported for *Pax3* mutant mice (dorsal neural tube, neural crest and myogenic defects). From these data, we conclude that expression of *Six4Δ* under *Pax3* regulation does not perturb normal embryonic development nor rescue *Pax3* deficiencies.

We next examined *Myod* expression in *Pax3^Six4Δ/+^* embryos using *in situ* hybridization. These experiments did not reveal any perturbation in *Myod* transcription (Figure 3E–3F). Comparison of *Myod* transcription in *Pax3* mutant embryos and in *Pax3^Six4Δ/Six4Δ^* homozygote embryos indicated that expression of *Six4Δ* does not prevent *Myod* expression and myogenesis when the *Myf5/Mrf4* myogenic pathway is active (Figure 3G–3H). The decreased expression of *Myod* observed in *Pax3^Six4Δ/Six4Δ^* embryos is similar to that observed in *Pax3^ILZ/ILZ^* embryos, as a result of cell death in the absence of Pax3 (data not shown).

### Expression of *Six4Δ in vivo* specifically impairs the *Pax3*-mediated myogenic pathway

**Figure 4:**
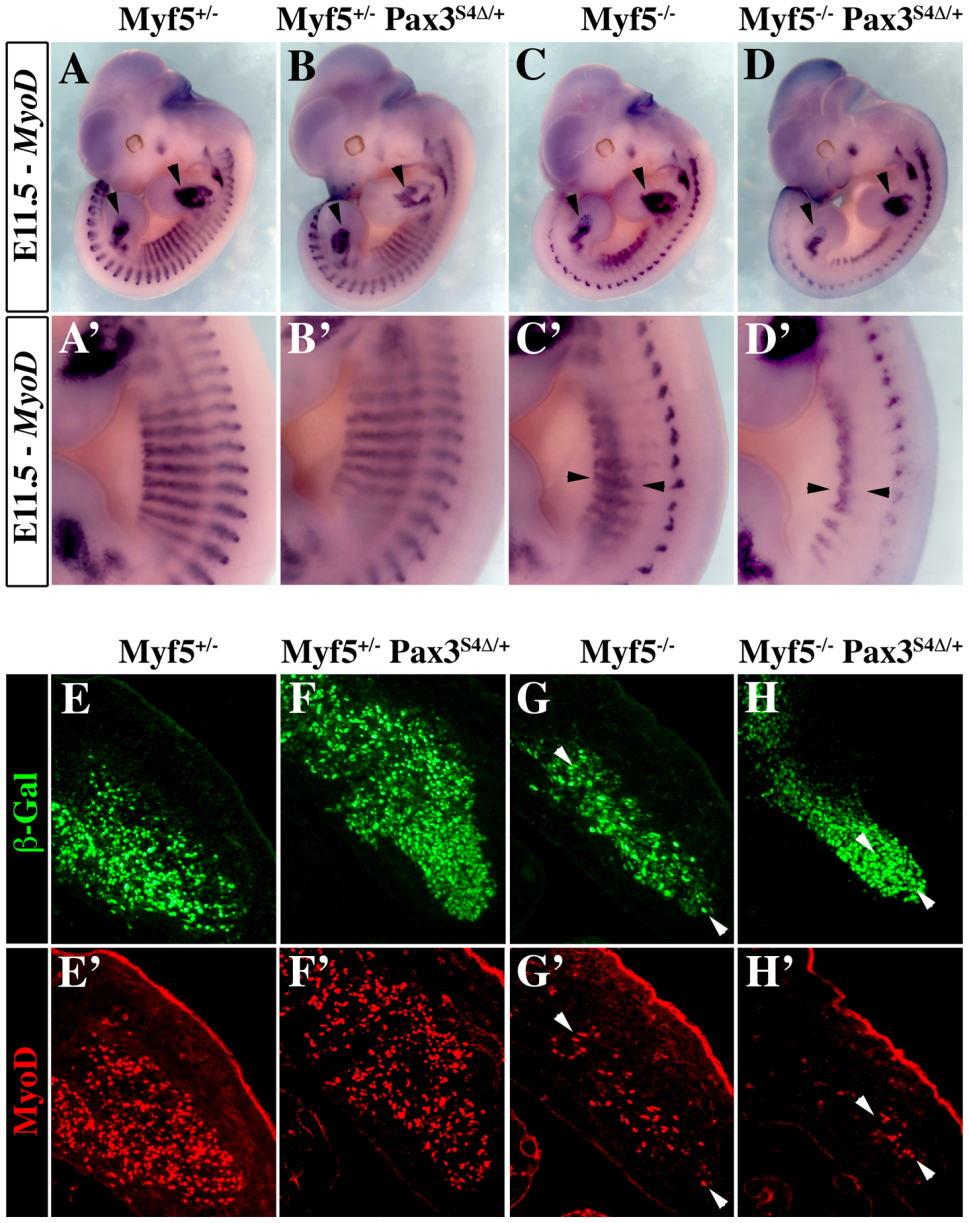
Six4Δ affects *Myod* expression and myogenesis in the absence of Myf5. A–D′, Whole mount *in situ* hybridization experiments using a *Myod* probe on *Myf5^+/−^* (A, A′), *Myf5^+/−^* : *Pax3^Six4Δ/+^* (B, B′), *Myf5^−/−^* (C, C′) and *Myf5^−/−^* : *Pax3^Six4Δ/+^* (D, D′) embryos at E11.5. At this stage, in *Myf5^−/−^* embryos (C, C′), *Myod* is activated and begins to rescue the formation of the myotome (arrowheads in C′). However, in *Myf5* deficient embryos which express *Six4Δ* under the control of *Pax3* regulatory elements, *Myf5^−/−^* : *Pax3^Six4Δ/+^* (D, D′), *Myod* expression is reduced, affecting the rescue of myotome formation (D′, arrowheads). In contrast, in thoracic somites of *Myf5^+/−^* : *Pax3^Six4Δ/+^* (B, B′) *Myod* expression is not altered compared to *Myf5^+/−^* embryos (A,A′). A′–D′, show enlargements in the interlimb region of A–D. E–H′, co-immunohistochemistry on transverse sections of hypaxial somites from *Myf5^+/−^* (E, E′), *Myf5^+/−^* : *Pax3^Six4Δ/+^* (F, F′), *Myf5^−/−^* (G, G′) and *Myf5^−/−^* : *Pax3^Six4Δ/+^* (H, H′) embryos at E11.5 using anti-β-Galactosidase (β-Gal) (green, E–H) and anti-Myod (red, E′–H′) antibodies confirms the severe reduction of Myod expression in *Myf5^−/−^* : *Pax3^Six4Δ/+^* (H, H′) embryos. Arrowheads indicate examples of cells in which the β-Gal reporter from the *Myf5^nLacZ^* allele is expressed and which co-express Myod.

**Figure 5:**
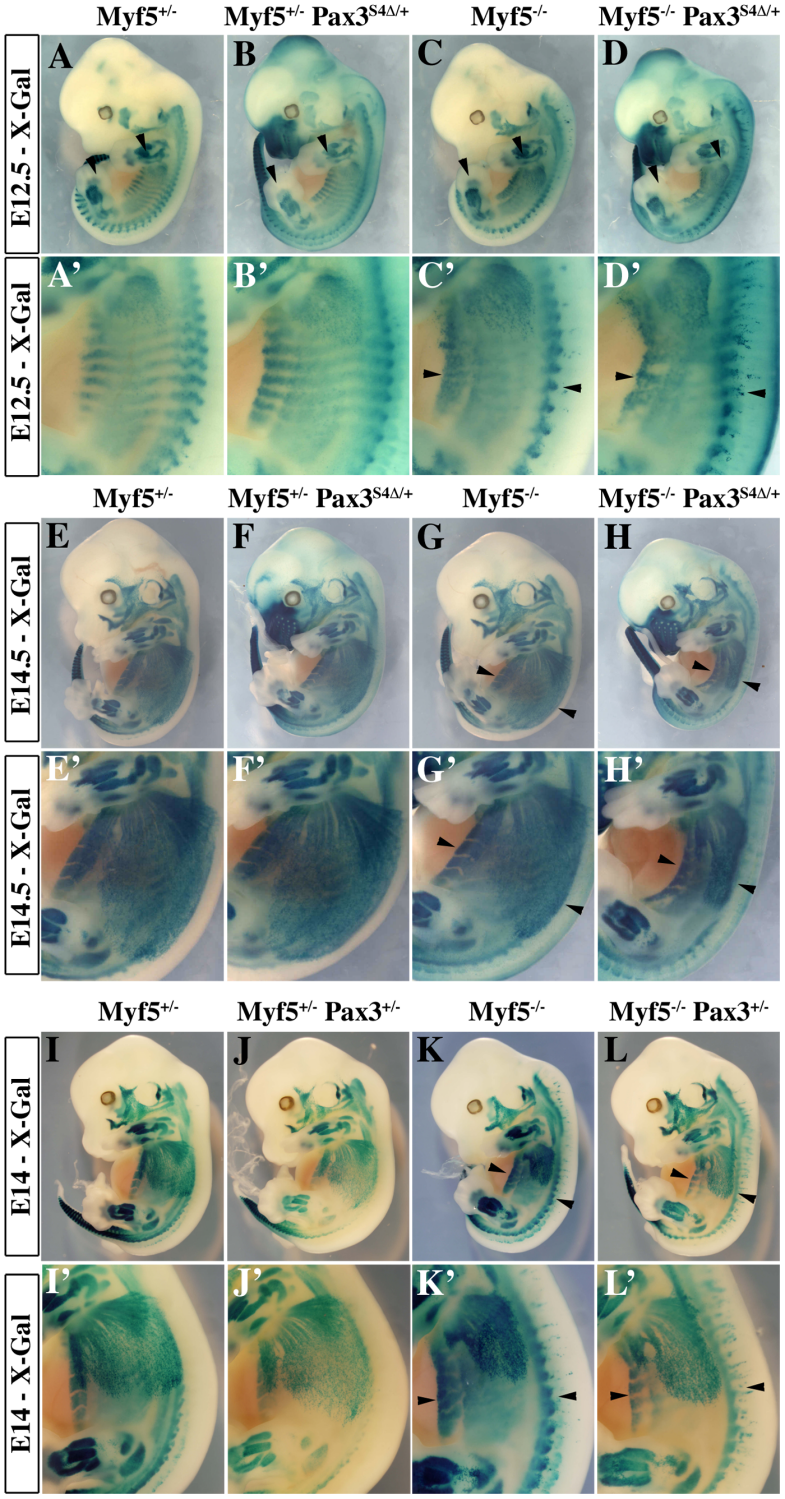
Impaired myogenesis in the presence of *Six4Δ*, in the absence of Myf5. A–L′; X-Gal staining of E12.5 (A–D′), E14.5 (E–H′) or E14 (I–L′) *Myf5^+/−^* (A, A′, E, E′, I, I′), *Myf5^+/−^* : *Pax3^Six4Δ/+^* (B, B′, F, F′), *Myf5^−/−^* (C, C′, G, G′,K, K′) and *Myf5^−/−^* : *Pax3^Six4Δ/+^* (D, D′, H, H′), *Myf5^+/−^* : *Pax3^+/−^* (J, J′), *Myf5^−/−^* : *Pax3^+/−^* (L, L′) embryos demonstrates that in *Myf5* deficient embryos which express *Six4Δ* under the control of *Pax3* regulatory elements, the localisation of myogenic cells, marked by the *Myf5^nLacZ^* reporter is impaired, notably in trunk muscles (H′ compared with L′). A′–D′, E′–H′, and I′–L′ are enlargements in the interlimb region of A–D, E–H and I–L respectively.

In order to determine if *Six-Eya* lies in the *Pax3*-*Myod* myogenic pathway, we crossed the *Pax3^Six4^* ^***Δ****/+*^ mice with *Myf5^nLacZ/+^* (abbreviated *Myf5^+/−^*) mice [33]. In wild type embryos, *Myod* expression is initiated around E10 in the hypaxial domain of thoracic somites [34]. However, in *Myf5* mutant embryos, *Myod* expression is delayed by about 24 h. Muscle formation is normal at later stages, indicating that Myod is able to rescue myotome formation in the absence of Myf5 after E11.5 [20]. In *Myf5^+/−^* and *Myf5^+/−^: Pax3^Six4^* ^***Δ****/+*^ embryos, *Myod* is activated normally and by E11.5 *Myod* expression is seen throughout the myotome (Figure 4A–4B, 4E–4F). As previously shown (Tajbakhsh et al., 1997), in *Myf5* mutant embryos (*Myf5^−/−^*, Figure 4C, 4G) *Myod* is activated later in the muscle precursor cells which are blocked in the epaxial and hypaxial somite, and at E11.5 the hypaxial part of the myotome is partially rescued (Figure 4G, arrowheads). In contrast, in *Myf5^−/−^: Pax3^Six4^* ^***Δ****/+*^ embryos, *Myod* expression is significantly reduced in epaxial and, notably, in hypaxial muscle precursor cells at E11.5 (Figure 4D, 4D′, 4H, 4H′). This impaired *Myod* expression leads to only partial rescue of myotome development; the cells that are expressing *Myod* in the hypaxial domain, despite activation of myogenic differentiation genes, like *Myogenin*, remain restricted to this part of the somite (data not shown). At E12.5, trunk muscles still show some disorganisation in *Myf5^−/−^* embryos, but this is more pronounced in *Myf5^−/−^: Pax3^Six4^* ^***Δ****/+*^ embryos (Figure 5A–5D, 5A′–5D′). By E14–E14.5, myogenesis is rescued in *Myf5^−/−^* fetuses (Figure 5G–5G′, 5K–5K′). In contrast, *Myf5^−/−^: Pax3^Six4^* ^***Δ****/+*^ fetuses display a reduction in trunk muscles (Figure 5H–5H′) which is more severe than in *Myf5^−/−^:Pax3^+/−^* embryos at this stage (Figure 5L–5L′). These results indicate that Six/Eya intervene in the Pax3-dependent pathway of *Myod* activation.

### *Myf5* is required for *Myod* activation in the absence of Six1 and Six4

**Figure 6:**
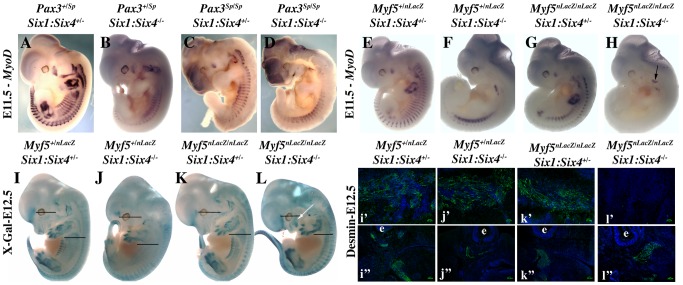
Axial *Myod* expression is lost in *Myf5^−/−^:Six1^−/−^/Six4^−/−^* embryos. A–H, Whole mount *in situ* hybridization using a *Myod* probe, I–L, X-Gal staining, and i′–l′, i″–l″ immunohistochemistry on sagittal sections of I–L embryos at the interlimb somites or head level using Desmin antibodies, at E11.5 (A–H) or E12.5 (I–L) with *Pax3^+/Sp^Six1^+/−^Six4^+/−^* (A), *Pax3^+/Sp^Six1^−/−^Six4^−/−^* (B) *Pax3^Sp/Sp^Six1^+/−^Six4^+/−^* (C), *Pax3^Sp/Sp^Six1^−/−^Six4^−/−^* (D), *Myf5^+/−^Six1^+/−^Six4^+/−^* (E, I), *Myf5^−/−^Six1^+/−^Six4^+/−^* (F, J) *Myf5^+/−^Six1^−/−^Six4^−/−^* (G, K), *Myf5^−/−^Six1^−/−^Six4^−/−^* (H, L) embryos, showing the role of Pax3/Six proteins and Myf5 acting upstream of *Myod* during trunk myogenesis. Desmin expression in E12.5 compound embryos at the axial level (i′–l′) and at the head level (i″–l″) is not detected in *Myf5^−/−^Six1^−/−^Six4^−/−^* embryos at the axial level (l′) but at the head level (l″), showing that craniofacial myogenesis can take place in this compound mutant. e: eye. White arrow in L shows the presence of craniofacial muscles.

We had previously shown that *Myod* expression is severely compromised in *Six1^−/−^/Six4^−/−^* double mutant embryos [13]. In these embryos, *Pax3* expression is maintained in anterior and posterior domains of the dermomyotomes, while impaired in the epaxial and hypaxial domains. Early *Myf5* expression is detectable, although decreased [13]. To test whether the remaining expression of *Pax3* and/or *Myf5* is responsible for the somitic activation of *Myod* observed in *Six* double mutant embryos, we examined *Myf5^−/−^:Six1^−/−^/Six4^−/−^* and *Pax3^sp/sp^:Six1^−/−^/Six4^−/−^* embryos. As shown in Figure 6, the expression of *Myod* is higher in *Pax3* mutants (*Pax3^sp/sp^*) compared to *Six1/Six4* double mutant embryos. *Myod* expression is still detectable, although decreased, in *Pax3^sp/sp^:Six1^−/−^/Six4^−/−^* embryos at E11.5. In contrast, *Myod* transcripts are not detectable in the somites of *Myf5^−/−^:Six1^−/−^/Six4^−/−^* embryos at E11.5 (Figure 6H), where *Myod* expression persists in the branchial arches (Figure 6H). X-Gal staining of compound *Myf5^−/−^:Six1^−/−^/Six4^−/−^* embryos at E12.5 further illustrates lack of axial myogenesis at a later stage (revealed by *Myf5-LacZ* and *Six1-LacZ*), while craniofacial musculature is still present (Figure 6L). Loss of trunk muscles is confirmed by desmin immunohistochemistry on E12.5 sections (Figure 6i′–6l′). The presence of myogenic desmin-positive cells in extra-ocular and masseter muscles (Figure 6i″–6l″, Figure S1) shows that craniofacial myogenesis is not abrogated in *Myf5^−/−^:Six1^−/−^/Six4^−/−^* embryos.

### Six proteins directly activate *Myod* regulatory elements

**Figure 7:**
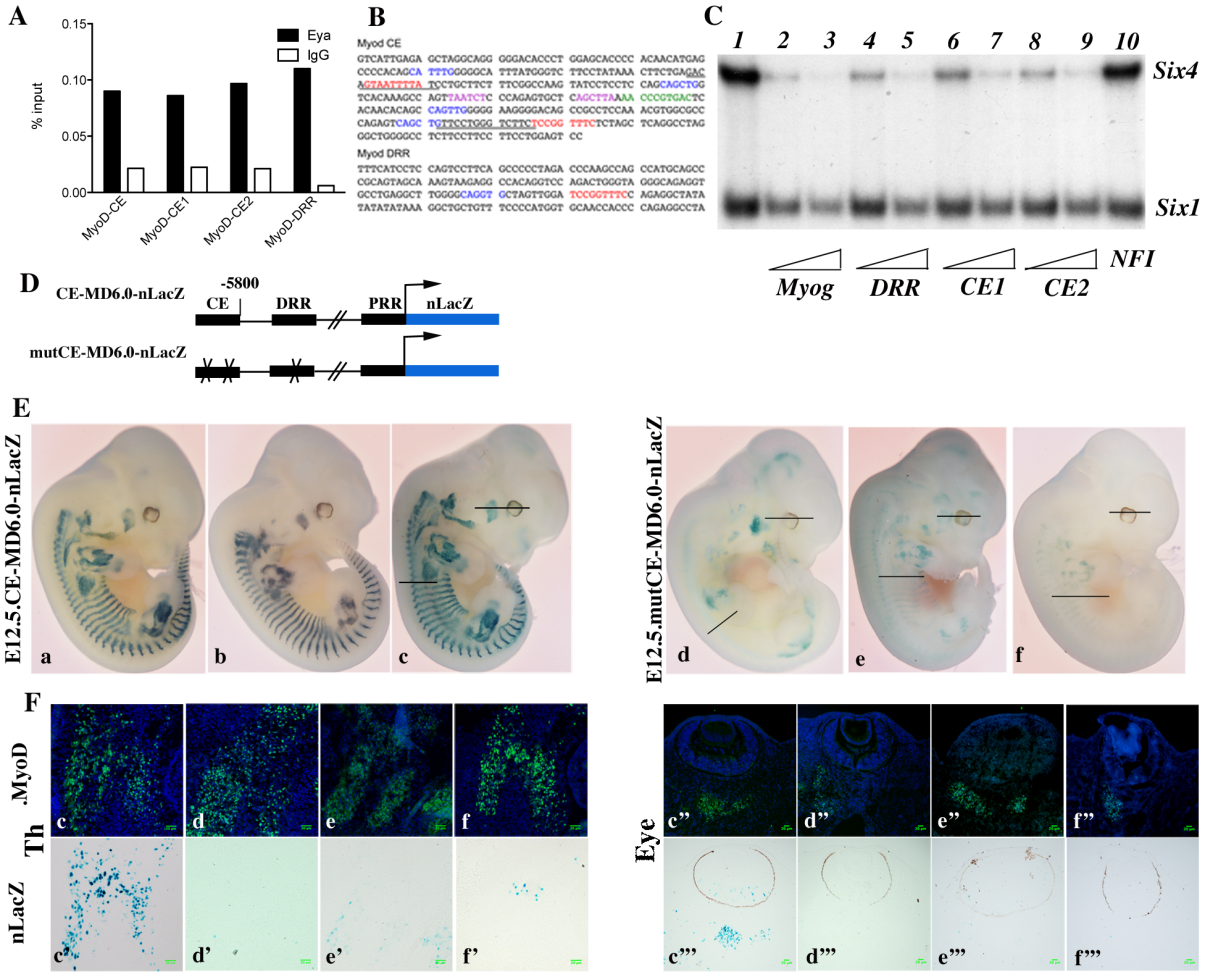
Six proteins are required for *Myod* expression in the mouse embryo. A- Chromatin Immunoprecipitation (ChIP) experiments performed with Eya antibodies or control IgG, on chromatin prepared from Pax3-GFP cells separated by flow cytometry from the trunk region of *Pax3^GFP/+^* embryos [32] at E11.5. ChIP experiments reveal association of Eya proteins with the core enhancer (CE) and distal regulatory region (DRR) 5′ of the *Myod* gene. B- Sequence of mouse *Myod* core enhancer (CE) and DRR. MEF3 sites are in red, E boxes in blue, Pitx sites in purple and Pax3 site in green. Underlined sequences correspond to the LS4 and LS15 linker-scanner mutagenesis performed on the human core enhancer [37]. C- Electromobility shift assays showing the interaction of Six1 and Six4 proteins with three distinct MEF3 DNA elements present in the regulatory regions of *Myod*. Radioactively labelled oligonucleotides with the *Myogenin* MEF3 site (Myog) were incubated with in vitro translated Six1 and Six4 proteins as a control (1). A 60 or 300 fold excess of unlabelled oligonucleotides containing the MEF3 *Myogenin* site (2,3), the MEF3 DRR site (4,5), the MEF3 CE1 site (6,7), the MEF3 CE2 site (8,9) or a 300 fold excess of unrelated *Myogenin* NFI oligonucleotides (10) were added in the reaction mix. D- Wild type and MEF3 mutant *Myod* transgenes used in the study, (not to scale). PRR, proximal regulatory region corresponding to the *Myod* promoter. E-Transient transgenic embryos with wild type or mutant *Myod* sequences at E12-E12.5. X-Gal staining of transgenic embryos with wt *CE-MD6.0-nLacZ* (a–c) or *mut3MEF3-CE-MD6.0-nLacZ* (d–f) transgenes. Six out of ten wild type transgenes expressed the *LacZ* reporter with the same expression pattern, three of them are shown. The number of transgenes inserted varied between 3 and 34 for X-Gal-positive (X-Gal+) embryos, and from 1 to 14 for X-Gal-negative (X-Gal−) embryos. Three out of eight mutant transgenic embryos expressed the *LacZ* reporter, all three are shown. The number of transgenes inserted was 23 (f), 39 (d) and 40 (e) for X-Gal+ embryos, and from 1 to 51 for X-Gal− embryos. F- Sections for one wild type (c) and for the three mutant transgenic embryos expressing the *LacZ* transgene were analysed for Myod protein by immunohistochemistry at the thoracic (Th) (c–f), and eye (c″–f″) levels to detect myogenic cells, thus revealing the % of transgene expression (X-Gal+ cells, c′–f′ and c′″–f′″) in the myogenic cell population (Myod-positive cells). While most Myod+ cells express the wt *Myod* transgene (c′, c′″), very few are marked by expression of the mutant *Myod* transgene (d′–f′, d′″–f′″).

*Myod* expression has been shown to be under the control of at least three separate DNA elements, a promoter region, a distal regulatory region (DRR), 6 kb 5′ of the transcription start site [35], and a core enhancer (CE) region, located 20 kb 5′ of the transcription start site (TSS) [36]. Both the CE and the DRR drive expression of a *LacZ* reporter to sites of myogenesis in transgenic embryos, where the CE shows a higher and more precocious activity [35], [37]. A specific deletion of either enhancer by the CREloxP system indicates functional redundancy [38], [39]. Both CE and DRR elements have been shown to bind Six1 and Six4 homeoproteins in growing and differentiating cells in the C2 muscle cell line [25]. Furthermore, both the CE and DRR are bound by Eya proteins *in vivo*, as shown by ChIP experiments on Pax3-GFP positive cells purified by flow cytometry from E11.5 embryos (Figure 7A and data not shown). One MEF3 element that binds Six proteins, is present in the DRR (5′TCcGGTTTC, which is conserved in the human sequence), and two in the CE (5′TaAaaTTaC, corresponding to part of the conserved box4 of the human sequence, shown to affect activity of the human enhancer in transgenic embryos [37], and 5′TCcGGTTTC, overlapping boxes 15 and 16 of the human CE sequence) (Figure 7B). These potential MEF3 sites bind Six1 and Six4 proteins, as shown in gel mobility shift experiments (Figure 7C). We next tested these sites for function in transgenic embryos. For these experiments we constructed a transgene in which the *Myod* CE sequence was inserted 5′ of the −5.8 kb flanking sequence of *Myod*, 340 bp upstream of the DRR element [35] to give a *CE-MD5.8-LacZ* transgene (Figure 7D). 6 out of 10 *CE-MD5.8-LacZ* transgenic embryos show X-Gal staining similar to that of endogenous *Myod* expression at E12.5 (Figure 7E and data not shown). Mutation of the three MEF3 sites compromised transgene activity, such that only 3 out of 8 transgenic embryos carrying the mutant sequences (*mut3MEF3-CE-MD5.8-LacZ*) show any *LacZ* expression at E12.5. In two of them, very low expression is detected in myogenic territories at the thoracic and limb levels (Figure 7E, 7Fe,e′,f,f′), while in the third (Figure 7E, 7Fd,d′, Figure S2) most of the *LacZ* transgene expression does not overlap with endogenous *Myod* expression. Expression in head muscles is detected with all wild type transgenes in Myod expressing cells (Figure 7E, 7Fc″,c′″, Figure S2). This is not the case with mutant transgenes, where most Myod expressing cells at the temporalis muscle or eye level do not express the mutant transgene (Figure 7E, 7F d″–f″; d′″–f′″, Figure S2). Sections of wild type and mutant embryos are shown in Figure 7F at trunk (left panels) and head (right panels) levels. Wild type *Myod* transgenes drive the expression of the *LacZ* reporter in 64 to 95% of Myod-positive cells, while mutant transgenes drive low expression of the *LacZ* reporter in 3 to 10% of Myod-positive cells. Expression is never detected in the tail somites in the posterior part of the embryo with the mutant transgene. Taken together, these experiments demonstrate a direct function of the Six binding sites in the activation of *Myod* during myogenesis in the embryo both in the trunk and head.

### Six2 is expressed in myogenic territories in the embryo

**Figure 8:**
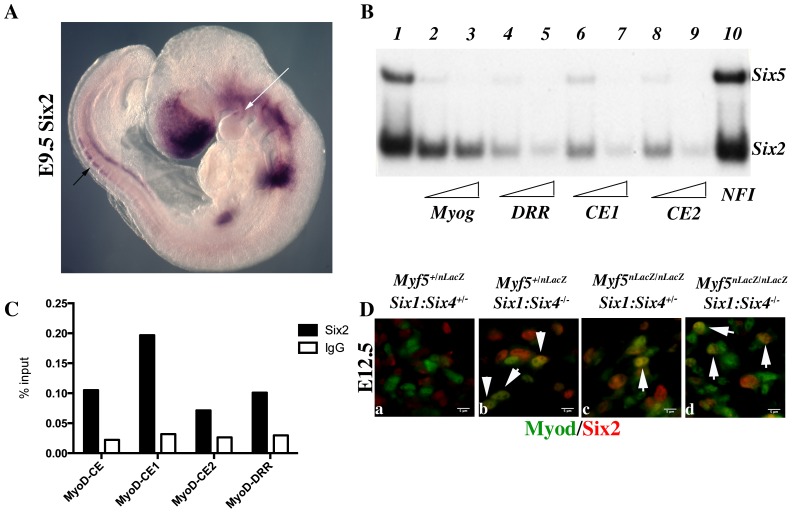
Six2 proteins bind *Myod* regulatory elements. Whole-mount in *situ* hybridization using a *Six2* probe on an E9.5 embryo. Note Six2 expression in dorsal aspects of newly formed somites (black arrow), and in the center of the first branchial arch (white arrow). B- Electromobility shift assays showing the interaction of Six2 and Six5 proteins with three distinct MEF3 DNA elements present in the regulatory regions of *Myod*. Radioactively labelled oligonucleotides with the *Myogenin* MEF3 site (Myog) were incubated with *in vitro* translated Six2 and Six5 proteins as a control (1). A 60 or 300 fold excess of unlabelled oligonucleotides containing the MEF3 *Myogenin* site (2,3), the MEF3 DRR site (4,5), the MEF3 CE1 site (6,7), the MEF3 CE2 site (8,9) or a 300 fold excess of unrelated *Myogenin* NFI oligonucleotides (10) were added in the reaction mix. C- Chromatin Immunoprecipitation (ChIP) experiments performed with Six2 antibodies or control IgG, on chromatin prepared from E12 *Six1−/−Six4−/−* embryos, and showing binding of Six2 *in vivo* on the regulatory elements of *Myod*. D- Immunocytochemistry performed with Six2 and Myod antibodies on E12.5 *Myf5+/−Six1+/−Six4+/−* (a), *Myf5+/−Six1−/−Six4−/−* (b) *Myf5−/−Six1+/−Six4+/−* (c), *Myf5−/−Six1−/−Six4−/−* embryos (d) at the masseter level, demonstrating Six2 (red) accumulation in Myod-positive (green) cells (white arrowheads).

To explain the discrepancies observed between *mut3MEF3-CE-MD5.8-LacZ* expression and the expression of *Myod* in *Six1/Six4* mutant embryos, we looked for other *Six* genes expressed in myogenic territories during embryogenesis [40] that could be responsible for the rescue of *Myod* expression observed in *Six1/Six4* embryos at the epaxial and craniofacial levels. *Six2* [41] and *Six5* [42], [43], [44] are the two other *Six* genes expressed in myogenic cells. By whole mount *in situ* hybridization, we further show that *Six2* is expressed in the first branchial arch of E9.5 embryos, and also in the dorsal regions of newly formed somites, where early epaxial *Myf5* is first activated (Figure 8A). Both Six2 and Six5 bind efficiently to the three *Myod* MEF3 elements, as determined by gel mobility shift experiments (Figure 8B). We next isolated chromatin for ChIP experiments to check if Six2 binds *in vivo* on *Myod* regulatory elements. With wild type embryos we did not observe significant binding. However with chromatin from E12 *Six1/Six4* mutant embryos we observed efficient binding of Six2 on *Myod* CE and DRR elements, demonstrating that Six2 can bind to *Myod* regulatory elements in the embryo (Figure 8C). We examined Six2 protein in the masseter muscle of *Six1^−/−^/Six4^−/−^* and *Myf5^−/−^:Six1^−/−^/Six4^−/−^* mutant embryos by immunocytochemistry at E12.5 and show that it co-localizes with Myod protein (Figure 8D). These results indicate that *Six2* is a good candidate for the activation of *Myod* expression in the absence of Six1 and Six4. They also suggest that *Six2* is upregulated under these conditions (Figure 8C, 8D).

## Discussion

We show that *Six1/4* play an essential role in the *Pax3/Myod* genetic pathway that regulates the onset of myogenesis [20]. This is revealed on a *Myf5* mutant background. Since the *Myf5* mutation that we use also affects *Mrf4*, entry into the myogenic program depends entirely on the myogenic determination factor Myod in the absence of Myf5/Mrf4 [45]. We illustrate with the Six4Δ sequence that Eya co-activators are required for Six transactivation, as previously shown [15], [30]. Furthermore our results show that key enhancer sequences of the *Myod* gene are directly regulated by MEF3 sites that are required *in vivo* at all sites of myogenesis to control *Myod* expression through the recruitment of Six1, Six2 and Six4 transcription factors.

The Pax/Six/Eya pathway to tissue specification is therefore important for the formation of skeletal muscle in the mouse embryo. However this network appears to be more complex than the *Eyeless/sine oculis/Eyes absent* cascade that leads to eye formation in *Drosophila* [1], [5]. As we show here for *Six1* and *Eya2*, their activation takes place in the absence of Pax3, whereas *Eyeless* initiates the cascade in *Drosophila*. In the mouse somite, Pax7 is also expressed in the central domain of the dermomyotome and may compensate. However, prior to the extensive cell death seen in the hypaxial somite in the absence of Pax3, *Six1/4* genes are transcribed. Furthermore during craniofacial myogenesis, the *Six1* gene and genes for Eya co-factors are expressed [41], [46] and the *polyMEF3-LacZ* reporter of Six transcriptional activity is high [15], in the absence of Pax3 that is not expressed during head myogenesis [5]. In *Six1^−/−^*/*Six4^−/−^* or *Eya1^−/−^*/*Eya2^−/−^* double mutants, *Pax3* expression is compromised in the hypaxial domain [15] indicating that Six/Eya can also regulate *Pax3*. Our analyses of *Six1^−/−^* and *Pax3^−/−^* mutants shows that they have overlapping but not identical myogenic phenotypes, confirmed by the double mutant phenotype which is more severe, particularly at later stages.

The Six4Δ sequence, which we targeted to an allele of *Pax3*, encodes a protein that still binds DNA, but does not bind Eya and is transcriptionally inactive, thus acting as a dominant-negative factor. The effectiveness of its action will depend on competition with wild type Six factors present in *Pax3* expressing cells. By diminishing the effects of Six factors (*Six2* and *Six5*, also expressed at sites of myogenesis [41] [42], as well as *Six1* and *Six4*), it serves as a probe, under conditions that are less radical than double mutants. This type of strategy, with a *Pax3^Pax3-En/+^* mouse line had previously proved valuable for probing Pax3 function [23]. In the absence of Myf5/Mrf4, when Six4Δ is present, down-regulation of *Myod* expression is clearly observed, under conditions in which somites are less perturbed, at E11.5–12.5. Later, the failure of skeletal muscles to develop leads to severe perturbations at sites of myogenesis in the trunk. Head musculature on the other hand appears normal, as do the forming limb muscles. In *Six1^−/−^/Six4^−/−^* double mutants, in the absence of Myf5/Mrf4, *Myod* is not transcribed in the trunk and limbs and myogenesis does not occur, whereas *Myod* transcripts are detectable in head muscle progenitors and muscle markers are present. These observations show that head myogenic progenitors, that are not derived from the somites, activate *Myod* and form head muscles in the absence of *Six1* and *Six4* [13]. This is in contrast to a report on zebrafish where *Six1a* was found to be essential for craniofacial myogenesis [16], and with reduced head myogenesis observed in compound *Six1;Eya1* mutant mice [17]. In addition to *Six1* and *Six4*, *Six2* and *Six5* genes are also transcribed in myogenic cells in the mouse embryo [41], [42], and we now show that Six2 can regulate *Myod* expression. Other transcriptional regulators, such as Pitx2, play an important upstream role in head myogenesis. Pitx2 has been shown to activate *Myod* in the trunk [47], where *Pitx2* lies genetically downstream of *Pax3*. However in the head, where Pax3 is not expressed at the onset of myogenesis [18], Pitx2 acts independently. In keeping with this, *Pax3/Mrf4/Myf5* triple mutants do not have defects in *Myod* activation and myogenesis in the head. However *Myod* is not activated in the trunk where skeletal muscles do not form [20]. In this context, *Six* genes do not rescue the phenotype.

Activation of *Myod* relies on two enhancer elements at 5 kb (DRR) and 20 kb (CE) upstream of the gene, as well as on the proximal promoter [35], [36]. In adult myogenic cells, Pax7 activates the promoter [48] and Pax3/7 have been shown to bind the CE in myogenic cell cultures [49], but there are no data on such a role of Pax3/7 in the embryo. The CE is an important regulator of embryonic *Myod* expression, but the DRR is also implicated in this activity. When the CE is deleted, delayed *Myod* expression is still observed, notably in the branchial arches and limb buds [39]. Deletion of the DRR does not abolish embryonic *Myod* expression [38], in keeping with the important role of the CE. We identify three separate DNA elements in the CE and DRR of *Myod* that are bound by Six1 and Six4 [25], and bound *in vivo* by Eya. In a transgene controlled by the proximal promoter, DRR and CE, we show expected expression of the *nLacZ* reporter at all sites of myogenesis in E12.5 embryos. When the Six/MEF3 binding sites are mutated, this activity is mainly lost, with low level expression at sites of myogenesis, in a few Myod-positive cells. These results show that Six transactivation is required for the function of these regulatory elements. Residual activity may be due to Myf5 activation of *Myod* regulatory sequences, through E-boxes that are also known to play an important role [37], [50]. Indeed our genetic experiments, which show that a major effect on *Myod* activation in the *Six1/4* double mutant is only seen in the absence of *Myf5*, are in keeping with this. In *Myf5/Mrf4* mutant embryos, Pax3-dependent rescue of CE enhancer activity is observed [39], potentially due to Six transactivation acting in the *Pax3/Six/Myod* pathway. In the linker scanning experiments where human *MYOD CE* elements were sequentially mutated [37], box 4 was found to be essential for expression in all skeletal muscle lineages. This sequence contains the Six binding site CE1. In contrast mutation of box 16 which contains our box CE2 did not lead to loss of activity, demonstrating that CE1 is the main functional MEF3 site [37].

In our transgenic analysis, mutation of Six/MEF3 sites leads to loss of transgene expression in most embryos, at sites of myogenesis in the head, as well as in the trunk. This contrasts with our findings with *Six1^−/−^/Six4^−/−^* and *Myf5^−/−^:Six1^−/−^/Six4^−/−^* mutants. An explanation for these discrepancies is that other Six proteins known to be expressed in myogenic cells compensate in some embryonic territories for the lack of Six1 and Six4. We provide evidence that Six2 may play such a role since it is expressed at myogenic sites (Figure 8A and [41]). In the *Six1^−/−^/Six4^−/−^* double mutant, Six2 is expressed in Myod-positive cells and binds the *Myod* regulatory elements. We have not been able to examine Six5 in this context due to the lack of appropriate antibodies.

**Figure 9:**
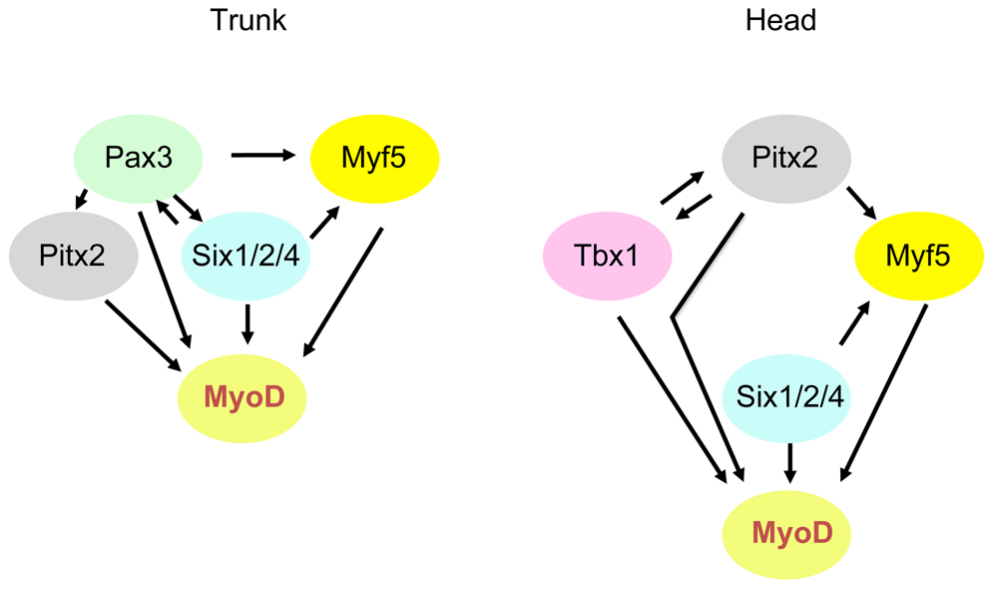
Schematic representation of genetic networks that activate *Myod* during myogenesis in the trunk and head. Variations in the interactions of factors in sub-domains at different developmental stages are not included here.

We conclude that during skeletal muscle formation in the trunk the *Pax3* genetic cascade that leads to *Myod* activation functions through *Six* genes and that in the absence of Myf5/Mrf4, the Six transactivation complex plays a key role in the activation of myogenesis. During the onset of craniofacial myogenesis, where *Pax* transcription factors do not play a role, *Six* expression is also a key determinant for *Myod* activation (Figure 9). Our analysis of the *Pax*/*Six*/*Eya* genetic cascade in the context of myogenesis has implications for the derivation of skeletal muscle from stem cell populations [44] and also, more generally, for other examples of tissue specification and organogenesis in vertebrates that also employ this genetic network [5].

## Materials and Methods

### Cloning, targeting vectors, and mice

*Six4* and *Six4Δ* cDNAs were obtained by screening a λgt11 library from adult mouse muscle (Clontech) [26]. The *Pax3^Six4Δ/+^* construct is derived from a construct previously reported [32]. The *Pax3^Six4Δ-ILZ)^* allele contains 2.4 kb of 5′ *Pax3* genomic region, in which the coding sequence of exon 1 is replaced by targeted sequences and 4 kb of 3′ sequence containing exons 2–4. The genomic sequences surround a Floxed *Puromycin* (*Puro)* cassette followed by 2.6 kb of *Six4Δ*cDNA, then an *IRESnLacZ* cassette. In addition, a *PGK*-*DTA* cassette encoding the A subunit of the *Diptheria* toxin gene (DTA) was inserted at the 5′ end of the construct to allow negative selection in ES cells. The targeting vector was electroporated into CK35 ES cells [51]. ES cells were selected and screened for recombination events by Southern blot analysis using *Eco*RV (RV in Figure 2) digests and a 5′-flanking probe (Figure 2E). Targeted ES cells were recovered with a 0.5–1% frequency and injected into blastocysts to generate chimaeras. Germline tramsmitted alleles were identified by the classical *Splotch* (*Pax3^Sp/+^*) heterozygote phenotype (lack of melanocyte colonization of the belly), and by PCR or by Southern blotting. *PGK*-*Cre* transgenic mice have previously been described [52]. *Six1^nLacZ/+^* mice, were crossed with *Pax3^Sp/+^* mice and X-Gal staining was performed as previously described [53]. *Six1^−/+^/Six4^−/+^* mice [13] were crossed with *Pax3^Sp/+^*or *Myf5^nLacZ/+^* mice [20].

All experiments with mice were performed according to the European Community Council Directive of 11/24/1986 (86/609/EEC) and with permission from the French Veterinary Services (permit number 75-1373) and approval by the Cochin General Animal Facility Service (accreditation number A-75-14-02). All efforts were made to minimize suffering.

### X-Gal staining, immunohistochemistry, and whole-mount *in situ* hybridization

We collected mouse embryos after natural overnight matings; for staging, embryonic day (E) 0.5 corresponded to midday assuming that fertilization had taken place at 6 a.m. Genotyping was carried out by X-Gal staining in X-Gal, with 0.2% PAF for 30 minutes following 1–2 h fixation in 4% PAF, on ice. When a light blue color had developed, embryos were rinsed in PBS and post-fixed overnight in 4% PAF. Whole mount *in situ* hybridization with digoxigenin-labelled riboprobes was performed as described [20]. The *Myod* riboprobe has also been previously described [20]. Fluoresencent co-immunohistochemistry was carried out according to [32], using the following antibodies : polyclonal anti-β-Gal (Molecular Probe, diluted 1∶200), monoclonal anti Myod (DAKO, 1∶200), monoclonal anti-Desmin (Abcam, 1/100), polyclonal anti Six2 (Proteintech, 1/200) and monoclonal anti-Myogenin (DAKO, 1∶200). Secondary antibodies were coupled to Alexa 488 1/250 and 546 1/1000 (Molecular Probes).

### DNA binding assays and transactivation

Gel mobility shift assays (GMSA) were performed essentially as previously described [26], with a labeled probe corresponding to the MEF3 site of the *Myogenin* promoter or *Myod* DRR and CE MEF3 elements and with Six1, Six2, Six4, Six5, Six4Δ and Eya2 proteins produced using the TNT T7 Coupled Reticulocyte Lysate System (Promega). The DNA templates used for *in vitro* transcription of mouse *Six1*, *Six2*, *Six4*, *Six4Δ, Six5* and *Eya2* were cloned in the pCR3 vector (Invitrogen). Because of the high molecular weight of Six4 and Six4Δ (about 90 kDa) we used a 3% acrylamide gel, run overnight at +4°C. To ensure that proteins were appropriately translated, parallel reactions were performed in the presence of [^35^S]methionine, separated on SDS-PAGE gels and visualized using autoradiography. The sequences of the double stranded oligonucleotides containing MEF3 sites used for bandshift assays are: DRR: 5′ AGT TGG ATC CGG TTT CCA GAG GC, CE1: 5′ TGA GAC AGT AAT TTT ATC CTG CT, CE2: 5′ GGT CTT CTC CGG TTT CTC TAG CT, *Myogenin* MEF3: 5′TGG GGG GGC TCA GGT TTC TGT GGC GT, *Myogenin* NFI: TAT CTC TGG GTT CAT GCC AGC AGG G. The TCAGGTTTC MEF3 sequence is underlined.

Chick primary myoblasts were grown and transfected as previously described [26], using RSV-Renilla as a control for transfection efficiency. Eya2, Six4 or Six4Δ expression was driven by the CMV promoter-enhancer present in pCR3, with the luciferase reporter gene under the control of a multimerized MEF3 element cloned upstream of the *human Aldolase A* minimal −35 to +45 bp promoter [54]. Two days after transfection, luciferase activity was measured using standard procedures.

### Generation and analysis of transient transgenic embryos

For the construction of the *CE-MD5.8-lacZ* and mutated *Mut3-CE-MD5.8-lacZ* sequences, mouse DNA was first used as a template to clone the core enhancer (CE) of *Myod* [36] with forward Apa1/Not1 5′ GGG-CCC-GCG-GCC-GCT-GAG-CCC-CAC-AGC-ATT-TGG and reverse 5′ GAA-TTC-CCC-CAG-CCC-TAG-GCC-TGA-GCT oligonucleotides; the MEF3 sequence is underlined. This 262 bp CE fragment was subsequently inserted into an Apa1-Pml1 site (position −5792 to−5652) of the *pMD6.8-lacZ* linearised plasmid [35], 340 bp upstream of the distal regulatory region (DRR) lying at −5310 bp from the *Myod* gene. The sequence of the *pCE-MD6.8-lacZ* reporter vector was verified by sequencing. To obtain mutated *pCE-MD6.8-lacZ*, one MEF3 site in the DRR (position −5176 to −5167) and two MEF3 sites in the CE (at position 55 and position 229) were mutated by substitution with a Hind III site (TCCGGTTTC->AAGCTTTTC), a XhoI site (GTAATTTTA ->CTCGAGTTA) or a BglII site (TCCGGTTTC->TCCAGATCT), respectively. The *pMutCE-MD6.8-lacZ* reporter vector was verified by sequencing. The plasmid was digested with Not1. Migration on an agarose gel allowed removal of plasmid sequences. Transgenic mice were generated by microinjection of the purified construct into fertilized F2 eggs from C57BL/6JxSJL mice, at a concentration of approximately 1 ng/µl using standard techniques. Injected eggs were reimplanted the same day or the day after the injection into outbred pseudo-pregnant foster mothers. Transient transgenic embryos were dated taking the day of reimplantation into the pseudo-pregnant foster mothers as E0.5. Embryos were dissected in PBS, fixed in 4% paraformaldehyde for 15 minutes, rinsed 3 times in PBS and stained in X-gal solution [33] at 37°C overnight. DNA was prepared from the vitelline membrane from each embryo and analysed by PCR, using *nlacZ* primers, and *Myod* primers in the DRR and in the CE.

### ChIP experiments

*Pax3^GFP/+^* males were crossed with C57Bl6N females to obtain *Pax3^GFP/+^* embryos. Somites were collected from E11.5 embryos by removing heads, neural tubes and internal organs. These samples were enzymatically digested with collagenase and dissociated cells were fixed with 1% formaldehyde at room temperature for 15 min. The GFP-positive cells were sorted by flow cytometry (BD FACs ARIA III). The gates for positive and negative GFP cells were determined using an equivalent sample isolated from wild type embryos and from *Pax3^GFP/+^* heterozygous embryos. About 7.5×10^5^ cells were collected for ChIP experiments from nine embryos. E12 *Six1^−/−^/Six4^−/−^* embryos were collected and enriched myogenic tissues were pooled after removing limbs, neural tube and internal organs. Dounce dissociated cells were fixed with 1% formaldehyde at room temperature for 15 min.

The chromatin immunoprecipitation procedure was performed according to the manufacturer's protocol (EZ-Magna ChIP G Kit; Merck Millipore) with antibodies recognizing all Eya proteins (Santa Cruz), Six2 protein (Proteintech), and Normal Mouse IgG provided in the EZ-Magna kit as a control. Input DNA and immunoprecipitated DNA were analyzed by quantitative-PCR (Roche, Light Cycler 480). Results were normalized with a negative control from an intergenic region without a MEF3 site (NC2). The sequences of primers were as follows:

*Myod-CE1* : Fwd : 5′ GGG CAT TTA TGG GTC TTC CT, Rev : 5′ GCC CTA GGC CTG AGC TAG A ; *Myod-CE4* : Fwd : 5′ GGG CAT TTA TGG GTC TTC CT, Rev : 5′ GCT GAG CAC TCT GGG AGA TT; *Myod-CE5* : Fwd : 5′ TCA GCT GTT CCT GGG TCT TC, Rev : 5′ GAC CTC TCA TGC CTG GTG TT; *Myod-CE7* : Fwd : 5′ AAC CCG TGA CTC ACA ACA CA, Rev : 5′ AGC CCT AGG CCT GAG CTA GA ; *Myod-DRR* : Fwd : 5′ GCC CGC AGT AGC AAA GTA AG, Rev : 5′ GCT CCC TTG GCT AGT CTT CC; NC2 : Fwd : 5′ GAG TTG GCA GGA ATC AGC TC, Rev : 5′ GCC AGC AAT TTG GTT TGA AT.

## Supporting Information

Figure S1 Immunohistochemistry with Desmin antibodies on sagittal sections of *Myf5^+/−^Six1^+/−^Six4^+/−^* (A), *Myf5^+/−^Six1^−/−^Six4^−/−^* (B), *Myf5^−/−^Six1^+/−^Six4^+/−^* (C), *Myf5^−/−^Six1^−/−^Six4^−/−^* (D) embryos at E12.5 at the masseter level, with DAPI staining.
(TIF)

Figure S2 Transient transgenic embryos with wild type or mutant *Myod* sequences at E12-E12.5. X-Gal staining of transgenic embryos with wt *CE-MD6.0-nLacZ* (c–c′″) or *mut3MEF3-CE-MD6.0-nLacZ* (d–d′″, e–e′″) transgenes, as presented in Figure 7E. Sections of wild type (c) and of two mutant transgenic embryos expressing the *LacZ* transgene were analysed for Myod protein by immunohistochemistry at the temporalis (c–e, c′–e′) and forelimb (c″–e″, c′″–e′″) levels to detect myogenic cells, thus revealing the % of transgene expression (X-Gal+cells, c′–e′ and c′″–e′″) in the myogenic cell population (Myod-positive cells). While most Myod+cells express the wt *Myod* transgene (c′, c′″), very few are marked by expression of the mutant *Myod* transgene (d′–e′, d′″–e′″).
(TIF)
