# Supplementary figures and images for: Six Homeoproteins Directly Activate Myod Expression in the Gene Regulatory Networks That Control Early Myogenesis
Source: PLoS Genet. 2013 Apr 25;9(4):e1003425. doi: 10.1371/journal.pgen.1003425 (PMC3636133; doi:10.1371/journal.pgen.1003425)

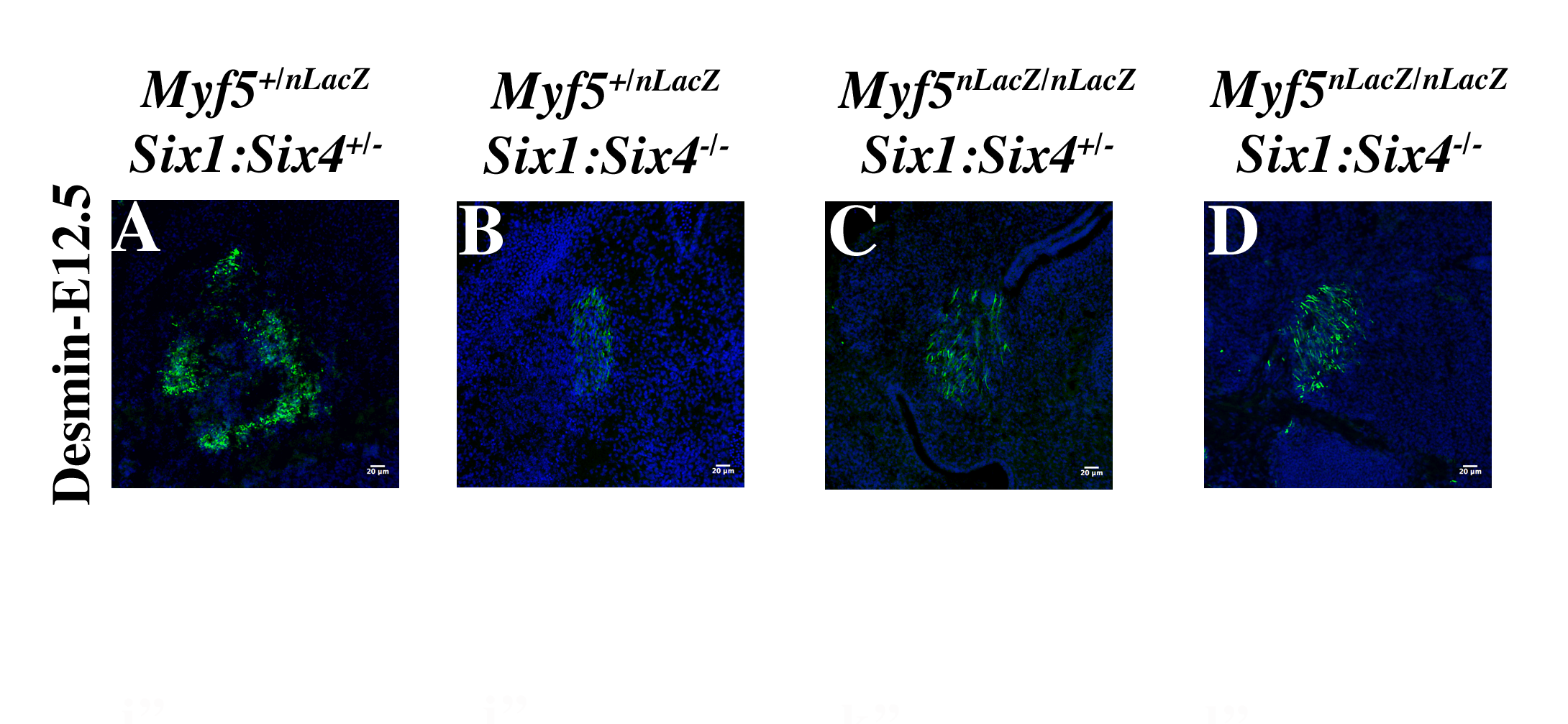

Supplement: Figure S1 — Immunohistochemistry with Desmin antibodies on sagittal sections of Myf5+/−Six1+/−Six4+/− (A), Myf5+/−Six1−/−Six4−/− (B), Myf5−/−Six1+/−Six4+/− (C), Myf5−/−Six1−/−Six4−/− (D) embryos at E12.5 at the masseter level, with DAPI staining. (TIF) [file pgen.1003425.s001.tif]

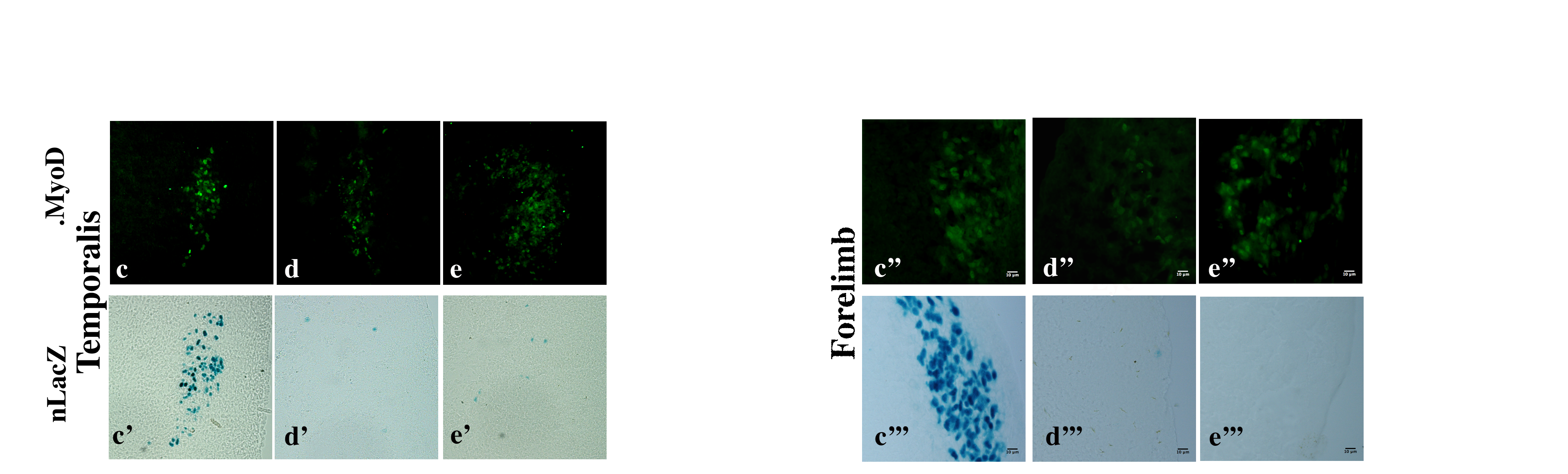

Supplement: Figure S2 — Transient transgenic embryos with wild type or mutant Myod sequences at E12-E12.5. X-Gal staining of transgenic embryos with wt CE-MD6.0-nLacZ (c–c′″) or mut3MEF3-CE-MD6.0-nLacZ (d–d′″, e–e′″) transgenes, as presented in Figure 7E. Sections of wild type (c) and of two mutant transgenic embryos expressing the LacZ transgene were analysed for Myod protein by immunohistochemistry at the temporalis (c–e, c′–e′) and forelimb (c″–e″, c′″–e′″) levels to detect myogenic cells, thus revealing the % of transgene expression (X-Gal+cells, c′–e′ and c′″–e′″) in the myogenic cell population (Myod-positive cells). While most Myod+cells express the wt Myod transgene (c′, c′″), very few are marked by expression of the mutant Myod transgene (d′–e′, d′″–e′″). (TIF) [file pgen.1003425.s002.tif]
